# Trends of population-based breast cancer survival in Germany and the US: Decreasing discrepancies, but persistent survival gap of elderly patients in Germany

**DOI:** 10.1186/1471-2407-12-317

**Published:** 2012-07-28

**Authors:** Bernd Holleczek, Hermann Brenner

**Affiliations:** 1 Division of Clinical Epidemiology and Aging Research, German Cancer Research Center, Im Neuenheimer Feld 581, 69120 Heidelberg, Germany; 2 Saarland Cancer Registry, Präsident Baltz-Straße 5, 66119 Saarbrücken, Germany

**Keywords:** Female breast cancer, Survival, Population-based, Trends, Germany, United States

## Abstract

**Background:**

Studies have revealed both higher cancer survival in the US than in Germany and substantial improvement of cancer survival in the past in these countries. This population-based study aims at comparing most recent 5-year relative survival of breast cancer patients and preceding trends in both countries.

**Methods:**

Women with a first invasive breast cancer diagnosed and followed up between 1988 and 2008 from Germany and the US (utilizing data from the Saarland Cancer Registry and the Surveillance, Epidemiology, and End Results Program, respectively) were included. Period analysis was used to derive most up-to-date 5-year relative survival and preceding survival trends according to age and stage.

**Results:**

Since 1993, age standardized relative survival has steadily improved in Germany and the US to 83% and 88%, respectively. In the period 2005–08, relative survival of localized cancer was above 97% in both countries, and 79% and 83% for locally/regionally spread breast cancer, respectively. Prognosis of metastasized disease has remained very poor overall, with improvement essentially being restricted to younger patients. The proportion of patients diagnosed with localized breast cancer was consistently higher in the US. If adjusted for stage, the differences in relative survival between both countries diminished over time and eventually disappeared.

**Conclusions:**

Similar survival is now observed in both countries for patients below the age of 70 years, but in Germany survival is still much lower for elderly patients. The observed trends point to treatment advances as a major cause for improved survival. However, substantial differences in mammography usage existed between both countries and might probably also account for the observed differences (to a lesser extent, also differences in health care systems, and delivery of cancer care). Encouraging, survival of breast cancer patients has improved in Germany to a much greater extent than in the US, albeit the persisting survival gap for elderly patients in Germany requires particular attention by researchers, public health authorities, and clinicians.

## Background

Population-based studies have shown a decrease in breast cancer (BRC) mortality during the past two decades in Germany and the US [1-3] primarily attributed to early detection activities and advances in cancer treatment [4,5] and demonstrated ongoing and substantial improvement of long-term survival of BRC patients [6-8]. Studies have also revealed higher BRC survival in the US than in Germany and other European countries [9-12].

Prolongation of cancer survival may have two principal causes: firstly anticipation of tumour diagnosis or even overdiagnosis by early detection and secondly increased survival due to improved effectiveness of cancer treatment. The time gained if a cancer is diagnosed earlier in a pre symptomatic stage due to screening is called lead time and artificially increases survival time. By itself, early detection does not affect the course of the disease. Therefore, screening requires treatment administered earlier to be effective and to postpone the death of the patient.

Overdiagnosis results from detection of malignant lesions that would never have progressed and presented clinically during the patient’s lifetime and survival analyses yield too optimistic estimates due to the inclusion of these non fatal cancers.

Therefore, comparative studies of population-based cancer survival should particularly take into account differences in stage distribution and early detection activities, to understand underlying mechanisms of observed trends over time or differences between populations.

Up to now, available comparative population-based studies on BRC survival were often limited as they provided survival trends stratified by age only (e.g. [6,7,9]) or reported stage-stratified survival only for singular calendar years [10,11].

A recently published study reported trends of BRC survival by age and stage in Germany [13]. This paper aims at comparing most recent 5-year survival of female BRC patients and preceding trends in Germany and the US by age and stage and discussing the results in the context of observed trends of BRC incidence and mortality, and available data on the implementation and usage of mammography screening and advances in BRC treatment in these countries in the past.

## Methods

### Database

For Germany, data from the population-based Saarland Cancer Registry were used. The registry covers the federal state of Saarland, Germany, with approximately 1.04 million residents in 2006 and operates since the beginning of the 1970s. Cancer notification is mandatory by law. The registry obtains notifications from pathology laboratories, hospitals, radiotherapy departments, outpatient clinics, and general practitioners. The completeness of case ascertainment is estimated above 95% [14,15].

The database from Saarland included 16,014 women aged 15 years or older diagnosed with invasive BRC (ICD-10 code: C50) between 1988 and 2008 (irrespective of previous primary cancers of other sites). Patients with a previous primary malignant tumour of the breast were excluded.

Mortality follow-up based on death certificates was available until end of 2008. Linkage with population registries was additionally performed for all patients assumed alive end of 2008 to identify patients lost to follow-up. Patients having migrated out of the Saarland region contributed survival time until removal. Patients for whom linkage with population registries was not possible (e.g. due to erroneous patient identifiers) were classified as "no follow-up available" and excluded from the analyses.

For the US, data from the Surveillance, Epidemiology, and End Results Program (SEER) were used and included the SEER-9 registries (states of Connecticut, Iowa, New Mexico, Utah, Hawaii, metropolitan regions of San Francisco-Oakland, Detroit, Atlanta, and Seattle Puget Sound area) from 1988 with extension to the SEER-13 registries (Alaska, regions of San Jose-Monterey, Los Angeles and Rural Georgia) from 1992 until 2008, covering a population of approximately 41.2 million residents in 2004–08 [16]. The survival analyses included 460,049 BRC patients based on criteria as above. Mortality follow-up was available until end of 2008.

The following data items were used: month and year of diagnosis, age at diagnosis, T classification, stage (categories: ‘localized’: T1-3N0M0 tumours; ‘local/regional spread’: T4M0 and N + M0 tumours; ‘distant metastasis’: any M1 tumours; unknown extent), basis of diagnosis, month and year of end of follow-up and vital status. Stage was classified according to summary stage reported by the registries or TNM information [17-19]. Clinical extent was used if no pathologic stage was available. For age-specific analyses, three age categories were used: 15–49, 50–69, and 70+ years. Patients with tumours notified from a death certificate only (DCO) were excluded from survival analyses, as were patients for whom no follow-up was available (Saarland only).

Additionally, incidence data of invasive and in situ BRC (ICD-10 codes: C50 and D05), mortality data (ICD-10 codes: C50), and population data were used from the registries.

The data used for this study are routinely collected by cancer registries according to state legislation for the purpose of monitoring cancer incidence, mortality, and outcomes and the anonymized data were used according to the respective provisions for the use of research data.

### Statistical analyses

Age-standardized rates (ASR) and truncated age-standardized rates (TASR) per 100,000 person years using the 2000 US standard population [20] were calculated for BRC incidence and mortality for successive calendar periods of four years between 1989 and 2008 in Germany and the US (all races combined). To account for differences in the registration of multiple primaries, for incidence estimation of invasive and in situ BRC, only the first cancer of the respective tumour type was considered.

A description of the BRC patients with regard to age, T classification, stage, basis of diagnosis, and DCO notification was provided for intervals of four calendar years between 1989 and 2008.

Relative survival (RS) which quantifies excess mortality due to the cancer (capturing both direct and indirect mortality) was derived as ratio of observed survival and expected survival for these persons derived from life tables of the underlying populations with respect to sex, age and calendar time (race was also considered for US patients) [21]. The Ederer II method was used to derive expected survival [22].

Life tables for Saarland were calculated for intervals of 5 years since 1990 from mortality and population data for single ages provided by the state statistical office (further details may be found elsewhere [13]). Region- and race-specific life tables for the US were derived from mortality and population data included the SEER database [23,24] for intervals of five calendar years since 1993 using the Elandt-Johnson method [25,26]. Race-specific life tables were either provided for white, black, and other races (three regions), white and races other than white (eight regions), and all races combined (two regions), according to the populations and numbers of deaths [27].

Period analysis was used to obtain most up-to-date estimates of RS. In addition to right censoring, observations are left truncated at the beginning of a calendar period [28]. Extensive empirical evaluation has shown that period estimates closely predict survival observed for the patients diagnosed in the respective calendar period [28-31]. In case of ongoing improvement, period analysis provides survival predictions as up-to-date as possible.

Period estimates of 5-year RS were derived for subsequent calendar periods of four years between 1993 and 2008. To avoid potential artefacts when combining classical cohort based estimates derived for calendar periods with complete follow-up and period estimates for the most recent calendar period, the period approach was continuously used for the entire study period.

To test for linear trend in RS over time, model based period analysis was employed. Poisson regression models for relative survival were fitted modelling the logarithm of the excess number of deaths with a linear predictor of follow-up year (categorical), age group (categorical) and calendar period (numerical), incorporating the logarithm of person time as offset [32]. A significance level of 0.05 (based on a two sided Wald test) was applied.

Besides crude survival, age standardized survival was derived as weighted average of age group-specific survival based on weights from the International Cancer Survival Standards (ICSS) [33]. Throughout the text, age standardized estimates are presented as overall survival. For both countries, stage information and T classification were reasonably complete (>70%) after 1988. Stage mix-adjusted survival was calculated using the stage distribution of the US patients diagnosed between 1988 and 2008. Standard errors were based on Greenwoods method [34]. The R Language and Environment for Statistical Computing (release 2.11.1; R Foundation for Statistical Computing, Vienna, 2011) and the add-on package ‘periodR’ (release 1.0-5) [35] were used for data preparation and analysis.

## Results

Table 1 shows trends of BRC incidence and mortality in Germany and the US. At the beginning of the 1990s, age standardized incidence of invasive BRC was higher in the US than in Germany but converged due to a decrease in the US among women aged 70+ years and an increase in Germany among women aged 50+ years to 113 and 111 new cases per 100,000, respectively, in 2005–08. Median age at diagnosis was lower in the US than in Germany (60 and 65 years, respectively).

**Table 1:** Breast cancer incidence and mortality in Germany and the US

		**Germany (Saarland)**					**US (SEER 9/13 regions)**^**b**^				
		**1989-92**	**1993-96**	**1997-00**	**2001-04**	**2005-08**	**1989-92**	**1993-96**	**1997-00**	**2001-04**	**2005-08**
incidence of invasive BRC	crude rate	115.7	128.8	140.7	153.2	162.9	118.2	115.8	124.3	120.0	118.7
	ASR ^a^	91.5	99.8	106.5	108.9	111.6	123.5	122.1	127.6	119.1	113.2
	15-49 (TASR ^a^)	56.1	56.2	59.1	53.5	57.5	60.7	57.1	58.1	57.2	57.7
	50-69 (TASR ^a^)	194.9	230.2	259.7	278.6	286.6	286.0	295.1	316.0	294.0	275.5
	70+ (TASR ^a^)	293.1	311.9	310.2	329.2	320.6	433.5	419.4	431.6	388.3	358.9
incidence of in situ BRC	crude rate	4.3	5.8	8.3	10.8	11.4	17.2	19.6	27.2	29.4	31.6
	ASR ^a^	3.8	4.9	6.7	8.4	8.5	18.6	21.1	28.3	29.4	30.3
	15-49 (TASR ^a^)	3.2	4.0	4.3	6.0	5.8	11.4	11.3	14.6	15.1	16.7
	50-69 (TASR ^a^)	7.9	11.9	19.8	23.4	22.6	47.9	57.0	77.2	80.7	79.7
	70+ (TASR ^a^)	7.7	7.7	9.6	11.7	14.9	42.7	52.5	72.1	74.3	76.6
BRC mortality	crude rate	44.1	47.4	50.3	48.2	49.3	30.0	28.6	26.0	24.9	24.7
	ASR ^a^	33.4	34.2	34.7	31.9	30.4	31.9	29.7	26.2	24.2	23.1
	15-49 (TASR ^a^)	13.2	12.5	11.7	8.8	7.1	11.0	9.7	8.3	7.4	7.0
	50-69 (TASR ^a^)	73.0	75.2	74.9	73.0	66.1	72.2	65.9	57.5	52.3	48.8
	70+ (TASR ^a^)	143.7	152.2	162.0	152.2	159.0	141.3	137.1	124.0	117.8	114.2

Incidence of invasive and in situ BRC (ICD-10 codes: C50/D05) and BRC mortality (ICD-10 code: C50) of women in Germany (Saarland) and the US (SEER 9/13 regions) between 1989 and 2008.

BRC: breast cancer; ASR: age standardized rate; TASR: truncated age standardized rate; rates given as new cases/deaths per 100,000; for incidence, only the first tumour of the respective type was considered; ^a^ the US2000 standard population was used for standardization; ^b^ the coverage of SEER program was extended from 9 to 13 registries in 1992.

Age standardized incidence of in situ BRC was about four-fold in the US compared to Germany (ASR: 30.3 vs. 8.5, respectively, in 2005–08). In the US, incidence among women aged 50+ years was similar and 5-fold compared to younger patients (TASR increased to 79.7 and 76.6 vs. 16.7, respectively, in 2005–08). In Germany, the incidence approximately tripled among patients aged 50–69 years (TASR: 22.6) and doubled among the other age groups (TASR: 5.8 and 14.9, respectively).

During the study period, a decrease of BRC mortality was observed in the US across all age categories (ASR: minus 28% to 23.1 in 2005–08), whereas in Germany mortality slightly decreased only among patients below 70 years of age but increased among older ones (ASR remained virtually unchanged: -3% to 30.4).

Table 2 presents characteristics of the included patients. The number of women diagnosed during each calendar period of four years has continuously increased. Most recently, the proportion of patients below 70 years of age was somewhat higher in the US than in Germany (71.5% vs. 65.6%, respectively). From 1989 through 2008, summary stage was available for 97.2% of the patients from the US, and for 81.6% of the German patients. The proportion of patients with T1 tumours and localized disease was continuously lower in Saarland than in the US (44.8% and 49.7% vs. 60.6% and 63.1%, respectively, in 2005–08). Almost all diagnoses were based on microscopic examinations (97.2% and 99.0%, respectively, in 2005–08). In the study period, the overall proportions of DCO notified BRC were 2.5% in Germany and 0.6% in the US, respectively.

**Table 2:** Characteristics of included breast cancer patients

**Category**		**1989-1992**		**1993-1996**		**1997-2000**		**2001-2004**		**2005-2008**	
		**n**	**%**	**n**	**%**	**n**	**%**	**n**	**%**	**n**	**%**
		Germany (Saarland)									
overall		2,564		2,877		3,118		3,354		3,494	
age	15-49 years	514	20.0	537	18.7	637	20.4	594	17.7	640	18.3
	50-69 years	1,179	46.0	1,322	46.0	1,418	45.5	1,591	47.4	1,653	47.3
	> = 70 years	871	34.0	1,018	35.4	1,063	34.1	1,169	34.9	1,201	34.4
stage	available	1,811	70.6	2,353	81.8	2,665	85.5	2,898	86.4	2,839	81.3
	localized ^a^	694	38.3	1,043	44.3	1,260	47.3	1,448	50.0	1,411	49.7
	local/regional spread ^a^	912	50.4	1,082	46.0	1,143	42.9	1,179	40.7	1,186	41.8
	distant metastasis ^a^	205	11.3	228	9.7	262	9.8	271	9.4	242	8.5
tumour size	available	2,013	78.5	2,513	87.3	2,872	92.1	3,120	93.0	3,127	89.5
	T1 ^a^	661	32.8	921	36.6	1,126	39.2	1,384	44.4	1,402	44.8
	T2 ^a^	953	47.3	1,116	44.4	1,249	43.5	1,249	40.0	1,293	41.3
	T3/4 ^a^	399	19.8	476	18.9	497	17.3	487	15.6	432	13.8
microscopic confirmation		2,418	94.3	2,761	96.0	3,018	96.8	3,251	96.9	3,397	97.2
death certificate only notified		82	3.2	62	2.2	52	1.7	59	1.8	79	2.3
no follow-up available		64	2.5	43	1.5	79	2.5	67	2.0	49	1.4
		US (SEER 9/13 regions ^b^)									
overall		64,556		87,228		97,713		97,579		98,857	
age	15-49 years	15,314	23.7	21,103	24.2	23,124	23.7	23,592	24.2	23,731	24.0
	50-69 years	27,007	41.8	36,641	42.0	42,501	43.5	44,147	45.2	46,914	47.5
	> = 70 years	22,235	34.4	29,484	33.8	32,088	32.8	29,840	30.6	28,212	28.5
stage	available	62,016	96.1	84,078	96.4	94,920	97.1	95,510	97.9	97,094	98.2
	localized ^a^	39,075	63.0	54,468	64.8	61,062	64.3	59,715	62.5	61,235	63.1
	local/regional spread ^a^	19,186	30.9	24,684	29.4	28,472	30.0	30,169	31.6	29,843	30.7
	distant metastasis ^a^	3,755	6.1	4,926	5.9	5,386	5.7	5,626	5.9	6,016	6.2
tumour size	available	53,027	82.1	73,352	84.1	85,692	87.7	87,682	89.9	93,254	94.3
	T1 ^a^	33,518	63.2	47,364	64.6	55,847	65.2	56,038	63.9	56,522	60.6
	T2 ^a^	14,848	28.0	20,080	27.4	22,746	26.5	24,007	27.4	27,088	29.0
	T3/4 ^a^	4,661	8.8	5,908	8.1	7,099	8.3	7,637	8.7	9,644	10.3
microscopic confirmation		63,443	98.3	85,855	98.4	96,357	98.6	96,364	98.8	97,831	99.0
death certificate only notified		394	0.6	497	0.6	602	0.6	554	0.6	479	0.5

Characteristics of female breast cancer patients (ICD-10 code: C50) from Germany (Saarland) and the US (SEER 9/13 regions) diagnosed between 1989 and 2008.

^a^ proportion among patients with available stage/T classification; ^b^ the coverage of SEER program was extended from 9 to 13 registries in 1992.

Trends of 5-year RS between 1993 and 2008 stratified by age group and stage are given in Table 3. Since 1993, overall age-standardized 5-year RS has steadily improved in both countries. Survival was much higher in the US than in Germany. However, the increase in 5-year RS was stronger in Germany (+10.3% units to 82.5% vs. +3.9% units to 88.1% in the US, respectively; p-values: <0.001), and the pre existing gap between both countries has diminished.

**Table 3:** Five-year relative survival of breast cancer patients by age and stage

**stage**		**Germany (Saarland)**										**US (SEER 9/13 regions**^**a**^**)**									
		**1993-96**		**1997-00**		**2001-04**		**2005-08**		**Δ**	**p-value**	**1993-96**		**1997-00**		**2001-04**		**2005-08**		**Δ**	**p-value**
		**RS**	**SE**	**RS**	**SE**	**RS**	**SE**	**RS**	**SE**			**RS**	**SE**	**RS**	**SE**	**RS**	**SE**	**RS**	**SE**		
overall	crude	73.0	1.1	77.1	1.0	80.1	0.9	84.2	0.9	11.2	<0.001	84.1	0.2	86.3	0.2	88.2	0.1	88.6	0.1	4.5	<0.001
	age std ^b^	72.2	1.3	76.3	1.2	79.0	1.1	82.5	1.0	10.3	<0.001	84.2	0.2	86.3	0.2	88.2	0.2	88.1	0.2	3.9	<0.001
	stage mix std ^c^	78.8	1.1	82.3	0.9	85.3	0.8	87.9	0.7	9.1	<0.001	84.1	0.2	85.7	0.1	87.7	0.1	88.1	0.1	4.0	<0.001
	15-49 years	75.4	2.0	78.6	1.8	83.9	1.6	87.7	1.4	12.3	<0.001	82.8	0.3	85.2	0.3	87.3	0.2	89.2	0.2	6.4	<0.001
	50-69 years	74.7	1.4	79.1	1.3	81.7	1.1	88.6	1.0	14.0	<0.001	85.4	0.2	87.5	0.2	89.8	0.2	90.0	0.2	4.7	<0.001
	15-69 years	74.9	1.2	78.9	1.0	82.3	0.9	88.4	0.8	13.5	<0.001	84.4	0.2	86.7	0.2	88.9	0.1	89.7	0.1	5.3	<0.001
	> = 70 years	69.3	2.4	73.6	2.2	75.1	2.1	74.9	2.1	5.6	0.026	83.6	0.4	85.5	0.4	86.5	0.4	85.7	0.4	2.1	0.001
localized	crude	90.7	1.5	93.3	1.2	96.0	1.0	97.5	0.9	6.8	0.002	95.4	0.2	96.1	0.2	97.0	0.2	97.2	0.1	1.8	<0.001
	age std ^b^	91.4	2.4	94.5	1.9	97.4	1.6	98.7	1.4	7.3	<0.001	95.7	0.2	96.3	0.2	97.3	0.2	97.3	0.2	1.6	<0.001
	15-49 years	86.9	2.5	92.3	1.9	93.1	1.6	95.3	1.4	8.4	0.011	93.6	0.3	94.1	0.2	95.4	0.2	96.3	0.2	2.7	<0.001
	50-69 years	91.7	1.8	91.6	1.5	95.4	1.2	98.7	0.9	7.0	0.001	95.4	0.2	96.4	0.2	97.4	0.2	97.6	0.2	2.2	<0.001
	15-69 years	90.1	1.5	91.8	1.2	94.7	0.9	97.8	0.7	7.7	<0.001	94.8	0.2	95.6	0.1	96.8	0.1	97.2	0.1	2.4	<0.001
	> = 70 years	93.2	4.6	98.3	3.5	99.9	2.9	96.6	2.9	3.4	0.555	96.7	0.5	97.0	0.4	97.5	0.4	97.4	0.4	0.7	0.290
	T1 age std ^b^	97.3	4.0	99.3	2.9	99.5	2.4	104.1	1.8	6.8	-	98.8	0.3	99.5	0.2	99.9	0.2	100.4	0.2	1.7	-
	T2-3 age std ^b^	89.4	3.7	92.1	2.9	93.9	2.4	92.1	2.4	2.7	0.110	86.3	0.6	87.0	0.5	89.7	0.5	89.3	0.5	3.0	<0.001
local/regional spread	crude	65.2	1.8	70.9	1.7	76.4	1.5	81.1	1.5	15.9	<0.001	75.8	0.4	79.0	0.3	83.3	0.3	85.0	0.3	9.2	<0.001
	age std ^b^	64.0	2.2	70.3	2.0	74.8	1.9	78.6	1.8	14.6	<0.001	75.1	0.4	77.5	0.4	81.7	0.4	82.8	0.4	7.6	<0.001
	15-49 years	70.7	3.4	71.4	3.1	81.1	2.7	82.6	2.6	11.9	0.004	75.5	0.6	80.2	0.5	83.5	0.4	86.8	0.4	11.3	<0.001
	50-69 years	65.3	2.5	72.2	2.2	77.6	1.9	86.2	1.7	20.8	<0.001	78.1	0.5	80.6	0.4	86.3	0.3	87.1	0.3	9.1	<0.001
	15-69 years	66.9	2.0	71.9	1.8	78.6	1.6	85.1	1.4	18.2	<0.001	76.9	0.4	80.4	0.3	85.2	0.3	87.0	0.2	10.0	<0.001
	> = 70 years	61.1	4.0	68.7	3.7	70.5	3.7	70.8	3.6	9.7	0.084	72.4	0.9	74.3	0.8	76.7	0.8	77.5	0.8	5.1	<0.001
	T1/2 age std ^b^	73.0	3.1	79.3	2.7	84.4	2.4	85.6	2.2	12.6	<0.001	81.4	0.5	83.6	0.5	87.7	0.4	88.1	0.4	6.7	<0.001
	T3/4 age std ^b^	47.1	3.6	52.3	3.5	56.2	3.4	64.3	3.2	17.2	<0.001	64.3	1.0	68.1	0.9	71.7	0.8	68.8	0.8	4.5	0.001
distant metastasis	crude	20.2	3.0	21.9	3.0	20.1	2.6	23.0	3.0	2.8	0.111	21.2	0.7	24.1	0.7	27.2	0.7	29.2	0.7	7.9	<0.001
	age std ^b^	20.3	3.2	22.1	3.0	19.0	2.6	23.7	3.0	3.3	0.106	20.2	0.7	22.5	0.7	25.2	0.7	26.7	0.7	6.5	<0.001
	15-49 years	11.5	5.8	17.9	8.9	26.9	7.6	29.6	9.1	18.2	0.029	26.8	1.5	31.5	1.4	34.5	1.4	38.8	1.4	11.9	<0.001
	50-69 years	25.3	4.4	24.7	4.4	23.6	4.0	29.1	4.6	3.8	0.237	21.3	1.0	24.4	1.0	29.0	1.0	30.5	1.0	9.2	<0.001
	15-69 years	22.0	3.7	23.9	3.9	24.5	3.5	28.5	4.1	6.5	0.058	23.2	0.9	26.9	0.8	30.9	0.8	33.2	0.8	10.0	<0.001
	> = 70 years	17.2	5.9	19.3	4.7	13.3	3.7	15.3	4.2	−1.9	0.712	17.4	1.1	18.2	1.1	19.0	1.1	19.4	1.1	2.1	0.045
unknown stage	crude	78.4	2.5	81.3	2.8	77.0	3.5	81.4	2.8	3.0	0.381	55.2	1.2	57.7	1.2	55.0	1.4	43.5	1.5	−11.7	<0.001
	age std ^b^	78.7	2.5	82.1	2.7	77.6	3.2	82.7	2.5	4.0	0.084	55.6	1.3	59.0	1.2	56.1	1.5	45.2	1.6	−10.4	<0.001

Five-year relative survival of female patients with invasive breast cancer (ICD-10 code: C50) from Germany (Saarland) and the US (SEER 9/13 regions) by age and stage for successive calendar periods.

RS: relative survival (period estimate) in %; SE: standard error; std: standardized; Δ : difference of RS between 2005–08 and 1993–96 in % units; ^a^ the coverage of SEER program was extended from 9 to 13 registries in 1992; ^b^ using weights from the International Cancer Survival Standards [33]; ^c^ weights derived from the SEER patients diagnosed 1988–2008.

In Germany, survival of patients aged 70+ years did not improve to the same extent as survival of younger patients (+5.6% units to 74.9% vs. +13.5% units to 88.4% in 2005–08; p-values: 0.026 and <0.001, respectively). In the US, survival of elderly patients improved to a lesser extent (+2.1% units to 85.7% vs. +5.3% to 89.7%; p-values: 0.001 and <0.001, respectively). As a result, a difference of about 11% units persisted in 5-year RS of elderly patients between Germany and the US.

Age standardized 5-year RS of localized BRC was 98.7% in Germany and 97.3% in the US in 2005–08. Five-year RS of BRC with local or regional spread was 78.6% and 82.8%, respectively. If distant sites were already involved at diagnosis time, 5-year RS was 23.7% and 26.7% in both countries, respectively.

Since 1993, most improvement of age standardized 5-year RS was observed in Germany for locally/regionally spread BRC, followed by localized disease (+14.6% units and +7.3% units, respectively; p-values: <0.001). At the same time, improvement of survival of US patients who already had much better prognosis in 1993–96, was less pronounced (+7.6% units and +1.6% units, respectively; p-values: <0.001) and the gap in 5-year RS between both countries reduced to 4.2% units and 1.4% units in 2005–08, respectively. Survival of distant BRC improved by 3.3% units (p-value: 0.106) in Germany and by 6.5% units in the US (p-value: <0.001).

Part of the overall survival differences between Germany and the US resulted from a higher proportion of patients with less advanced BRC in the US. Adjustment for stage mix reduced survival differences between both countries. In 2005–08, these differences eventually had disappeared (87.9% and 88.1%, respectively).

Differences in most recent stage-specific 5-year RS and preceding trends between patients aged > = 70 years and younger ones were varying. Survival of patients with localized BRC was equally high among elderly and younger patients (in 2005–08, it was 96.6% vs. 97.8% and 97.4% vs. 97.2%, respectively, in Germany and the US). Survival of patients with locally/regionally spread BRC improved less and was much lower for elderly patients in both countries (in 2005–08, it was 70.8% vs. 85.1% and 77.5% vs. 87.0%, respectively). Among patients with metastasized BRC, improvements were highest for patients <50 years of age (+18.2% units and +11.9% units to 29.6% and 38.8%, respectively; p-values: 0.029 and <0.001), but no substantial improvement was seen for elderly patients (changes of −1.8% units and +2.1% units to 15.3% and 19.4%, respectively; p-values: 0.712 and 0.045).

Age standardized 5-year RS of BRC patients with unknown or missing stage information was varying in both countries. In Germany, it was about 80% during the study period. In the US, it varied slightly between 55% and 58% between 1993 and 2004, but dropped to 45% in the most recent calendar period.

## Discussion

In this study, most recent 5-year RS of female BRC patients from Germany and the US and preceding trends were analyzed using data from the population-based Saarland Cancer Registry and the SEER 9/13 registries. In 2005–08, age standardized 5-year RS was 82.5% and 88.1% for all stages combined, 98.7% and 97.3% for localized BRC, 78.6% and 82.8% for BRC with local or regional spread and 23.7% and 26.7% for tumours with involvement of distant sites at diagnosis, respectively, in Germany and the US.

During the study period, most overall improvement of 5-year RS was observed for locally/regionally spread BRC, followed by localized tumours. As increases were more pronounced among German patients, the existing discrepancies between the two countries have decreased over time. Among patients with metastasized BRC, substantial improvement was seen only for patients below 50 years of age. The differences in stage-adjusted survival have entirely disappeared over time.

In Germany, 5-year RS and its improvement over time were inferior for patients aged 70+ years resulting both in an increased age gradient in Germany and prevailing differences in 5-year RS of elderly patients between the two countries.

The health care systems differ fundamentally in both countries. In Germany, health care is decentralized with private practice physicians and mostly public hospitals providing most ambulatory and impatient care. In 2007, 95.7% of the population had health insurance coverage through public or private health insurance [36]. In the US, health care facilities largely belong to the private sector and health insurance by private or government health insurance schemes covered 84.7% of the population in 2006/07 [37]. Both countries have a high standard of living and spend more than 10% of their gross domestic product into health care [38].

In the US, organized breast cancer screening was introduced (with regional differences and different types of programs) at the beginning of the 1990s [39]. Widespread use of mammography has been reported. The proportion of women aged 50 years or older reporting to have had a mammogram within the past two years increased between 1987 and 2005 from 27.4% to 68.4% [40,41].

In Germany, implementation of organized mammography screening started only recently in 2003. The quality-assured programme offers women aged 50 to 69 years a screening mammography every two years. Prior to its introduction in 2006, only opportunistic screening was available in Saarland [42]. In 2002–04, 45.3% of women aged 55 years or older residing in Saarland reported to have had a mammography within the last two years (unpublished data from the population-based ESTHER cohort study in Saarland [43,44]). For 2007–08, a proportion of 53% of eligible women participating in the organized screening was reported [45,46].

In the US, organized mammography screening started earlier and was intensely used even among younger and elderly women. Data on the usage of opportunistic mammography from Germany prior to the introduction of the organized screening are sparse. Based on the data available, it may be assumed that mammography usage and screening was much lower in Germany during the study among all age categories.

Higher usage of mammography in the US compared to Germany is well reflected in the presented incidence data. Higher proportions of earlier stages and a fourfold incidence of in situ tumours in the US correlate with the differences in the adoption and intensity of mammography screening [47-49].

Diagnosis of in situ lesions of the breast is usually based on a microscopic examination and basis for a notification from a pathology laboratory. According to the registration practises of the SEER registries and the Saarland Cancer registry, we assumed the observed differences in incidence of in situ tumours to represent differences in screening activities (or prevalence of risk factors) rather than differences in case ascertainment.

The observed drop in incidence of invasive BRC in the US after the year 2000 may reflect the change in the prescription of hormone replacement therapy [50,51], whereas the implementation of mammography screening probably interacts with this effect in Germany. However, differences and changes in the prevalence of ‘traditional’ risk factors have also to be kept in mind. BRC mortality started to decline at the end of the 1980s in the US and approximately ten years later in Germany (data not shown).

To account for overestimation of survival of patients with screen detected cancers we performed analyses stratified by age and stage. This at least partly allowed combining patients with comparable stages with regard to begin of follow up, even if lead time effects remain present within all stages. Furthermore, beneficial effects of screening have even been demonstrated between clinically and screening detected tumours of same stage [52].

The introduction of organized screening in Germany at the end of the study period, the higher intensity of mammography usage in the US on the one hand, and a much higher increase in 5-year RS in Germany on the other hand, support the presumption of advances in the treatment of the disease and adoption of treatment recommendations as a major cause of the remarkable gain in survival in Germany and advanced disease during the study period. However, increased early detection surely has interfered with these developments. As an observational study, it cannot fully resolve the contributions of improved treatment and screening as underlying reasons of the striking increase of 5-year RS in Germany.

Major advances in BRC treatment during the past 20 years included improved staging (e.g. more sensitive medical imaging and sentinel node dissection), propagation of breast conserving surgery and adjuvant radiotherapy for effective local treatment of early stage disease and new agents for chemotherapy and antiestrogen treatment, and targeted biologic agents. Treatment according to consensus recommendations and tailored to the patient's individual risk and a wider range of treatment options has increased the number of patients eligible for cancer-specific treatments.

For elderly patients, co-morbidity and differences in the delivery of cancer care are well documented (e.g. delay in seeking cancer care and influences of co-morbidity on selection of cancer treatments [53], lower likelihood of receiving adjuvant radiotherapy or chemotherapy compared to younger patients, even after adjustment for co-morbidity and extent of disease [54-57]) and may explain inferior 5-year RS and its less pronounced increase [58-60].

The widespread use of mammography among elderly women in the US (the proportion of women aged 75+ years reporting to have had a mammogram within the last two years increased from 17.3% to 54.7% between 1987 and 2005) [41] most likely exaggerated the increase in survival over time among these patients and may have partly concealed existing differences in age-specific survival in the US, as lead time effects and overdiagnosis particularly strongly affect survival estimates of a population segment with increased (age-related) mortality. Nevertheless, the observed differences in the survival of elderly patients between both countries – particularly pronounced in locally/regionally advanced disease – may point to possible limitations and hesitant delivery of care to elderly patients in Germany.

In the interpretation of the findings, a number of limitations and strengths should be considered. A major limitation is the restricted availability of clinical information. Although rather crude, the used staging scheme allowed survival estimation for three rather distinct groups of BRC patients with regard to available treatment options. Tumour stage and T classification were sufficiently complete.

The proportions of patients with missing stage were much higher in Germany than in the US. Survival of German patients with unknown stage was comparable to overall survival. Survival of US patients with unknown stage was much lower, possibly due to higher proportions of patients with clinical cancers of more advanced stage.

Further determinants beyond age and tumour stage, e.g. information about administered treatments, socio-economic status, or co-morbidity were not available. Neither was the method of first detection to consider screening effects as well as lead time, length time and overdiagnosis [5,61].

The included registries operate as established registries with high levels of completeness of case ascertainment and follow-up [14-16,62]. So far, no other population-based cancer registry from Germany may provide trend data as presented in this article. Even if it constitutes only 1.3% of the overall population, the Saarland region is well representative for Germany and its health care system and the size of its population allowed analyses stratified by age and stage as most fundamental prognostic factors with sufficient precision. However, when interpreting point estimates of 5-year RS we also considered the corresponding trends, particularly for strata with small numbers of subjects.

The 17 registries currently included in the SEER programme cover approximately one fourth of the US population. The covered regions are regarded reasonably representative for the US, although the population is categorized as somewhat more urban, with less unemployment, higher education and higher smoking prevalence [16,63,64]. One has to keep in mind, that such population differences might also have affected participation in mammography screening and access to cancer care of the included SEER populations [65,66].

To avoid artefacts in the most recent survival trends due to the inclusion of the four registries which joined the SEER programme in 2000 (California regions, Kentucky, Louisiana and New Jersey), we restricted the analyses to the SEER 13 regions. Additionally performed analyses of these four regions provided almost identical survival estimates for 2005–08 (differences of overall, age- and stage-specific estimates ranging between 0.2 and 1.7% units; data not shown).

The validity of cancer diagnoses may be considered high, as almost all tumours were microscopically confirmed. When interpreting stage-specific survival, stage migration (a shift of classification towards more advanced stages due to increased sensitivity and improved accuracy of diagnostic procedures) has to be kept in mind [67]. Interim revisions of the BRC classification schemes [17-19] might be of little effect only with regard to the used clinical stages.

In addition to comparability issues of included patients and tumour stages, the completeness of follow-up and estimation of background mortality is crucial in survival comparisons.

The proportions of DCO notified cases were rather small in the used databases. Under such circumstances, the effects of the exclusion of DCO cases from the analysis or a possible correction for DCO cases are expected to be very small according to recent work [68].

RS as a measure for survival in the hypothetical situation where the disease under study would be the only cause of death (denoted as net survival) is dependent on the general population mortality (the composition of cancer patients under follow-up changes over time with regard to demographic characteristics) [69]. To account for such dependencies and minimize effects we provided and used age standardized estimates for inter country comparison.

The strategy of using the period analysis approach for all subsequent calendar periods aimed at deriving most up-to-date estimates (even in case of ongoing improvement) and consistent use of methodology to prevent potential artefacts in the derived time trends due to a mixture of classical cohort based and period survival estimates.

The observed trends of RS and corresponding p-values were considered on their own and carefully interpreted in the light of further empirical observations. Therefore, we did not account for multiple testing despite the overall number of performed statistical tests for significance [70].

Follow-up was almost complete for both the German and the US patients. It has been shown, that national life tables may not adequately describe overall mortality of a subpopulation and result in biased estimates of RS (e.g. for the US, use of national life tables overestimates SEER survival estimates) [71]. Therefore, region- and race-specific life tables were derived for the analyses.

As the aim of population-based survival analyses is to provide a picture of the overall control of cancer in the societies compared, we did not restrict the analyses to any ethnic subpopulation (e.g. inclusion of white US patients only). Otherwise this would result in an inappropriate selective exclusion of socio-economically less privileged patients in one of the countries compared. Additional analyses were performed with a restriction to white US patients only. The effects of such an exclusion were generally small (estimates of crude, age standardized and stage adjusted survival estimates of whites were 1.5% units above estimates of all races combined), but considerable effects were observed for younger patients and more advanced stages (e.g. survival of whites aged 15–49 years with metastasized BRC was 4.5% units higher than estimates of all races combined; data not shown).

## Conclusions

The findings may have several important implications. Transatlantic differences in 5-year RS and tumour related excess mortality of BRC patients revealed in previous studies have continuously decreased in recent years.

The observed survival trends point to advances in BRC treatment as a major cause for the gain in prognosis in both countries. Differences in early detection activities and effects due to lead time and overdiagnosis further account for the observed differences.

Whereas most overall improvement was observed for patients with locally or regionally advanced disease, prognosis of patients with distant sites involved at diagnosis is still limited. Despite interim advances in the availability of (increasingly costly) treatment options for patients with metastasized disease, only some moderate overall improvement has been observed for these patients during the past 15 years. The implementation of mammography screening in Germany in recent years will hopefully further reduce the numbers of breast tumours with advanced stage.

Particularly for Germany, this study indicated striking deficits in cancer survival of elderly patients. The gap in cancer related mortality between elderly patients and younger ones in Germany has even increased over time. Inequalities in the access to and provision of cancer care of elderly patients need to be jointly focussed on in future by cancer research, health care policy, and the medical community.

## Abbreviations

BRC: Breast cancer; SEER: Surveillance, Epidemiology, and End Results Program; ASR: Age-standardized rate; TASR: Truncated age-standardized rate; DCO: Death certificate only; RS: Relative survival; ICSS: International Cancer Survival Standards.

## Competing interests

The authors declare that they have no competing interests.

## Authors’ contributions

This study was designed by BH and HB. BH prepared and managed the dataset and analyzed the data. BH and HB provided interpretation of the results. BH drafted the manuscript and HB critically reviewed and revised the draft. Both authors have approved the final version of the manuscript.

## Pre-publication history

The pre-publication history for this paper can be accessed here:

http://www.biomedcentral.com/1471-2407/12/317/prepub
